# Tracing coco de mer's reproductive history: Pollen and nutrient limitations reduce fecundity

**DOI:** 10.1002/ece3.3312

**Published:** 2017-08-24

**Authors:** Emma J. Morgan, Christopher N. Kaiser‐Bunbury, Peter J. Edwards, Frauke Fleischer‐Dogley, Chris J. Kettle

**Affiliations:** ^1^ ITES–Ecosystem Management ETH Zürich Zürich Switzerland; ^2^ Ecological Networks Department of Biology TU Darmstadt Darmstadt Germany; ^3^ Singapore‐ETH Centre Singapore City Singapore; ^4^ Seychelles Islands Foundation Victoria Mahé Seychelles

**Keywords:** flower production, fruit abortion, fruit set, habitat degradation, *Lodoicea maldivica*, nutrient allocation, pollen limitation, reproductive ecology, seed size, Seychelles Islands

## Abstract

Habitat degradation can reduce or even prevent the reproduction of previously abundant plant species. To develop appropriate management strategies, we need to understand the reasons for reduced recruitment in degraded ecosystems. The dioecious coco de mer palm (*Lodoicea maldivica*) produces by far the largest seeds of any plant. It is a keystone species in an ancient palm forest that occurs only on two small islands in the Seychelles, yet contemporary rates of seed production are low, especially in fragmented populations. We developed a method to infer the recent reproductive history of female trees from morphological evidence present on their inflorescences. We then applied this method to investigate the effects of habitat disturbance and soil nutrient conditions on flower and fruit production. The 57 female trees in our sample showed a 19.5‐fold variation in flower production among individuals over a seven‐year period. Only 77.2% of trees bore developing fruits (or had recently shed fruits), with the number per tree ranging from zero to 43. Flower production was positively correlated with concentrations of available soil nitrogen and potassium and did not differ significantly between closed and degraded habitat. Fruiting success was positively correlated with pollen availability, as measured by numbers and distance of neighboring male trees. Fruit set was lower in degraded habitat than in closed forest, while the proportion of abnormal fruits that failed to develop was higher in degraded habitat. Seed size recorded for a large sample of seeds collected by forest wardens varied widely, with fresh weights ranging from 1 to 18 kg. *Synthesis*: Shortages of both nutrients and pollen appear to limit seed production of *Lodoicea* in its natural habitat, with these factors affecting different stages of the reproductive process. Flower production varies widely amongst trees, while seed production is especially low in degraded habitat. The size of seeds is also very variable. We discuss the implications of these findings for managing this ecologically and economically important species.

## 1 INTRODUCTION

Numerous biotic and abiotic factors acting at various stages in the lifecycle influence a plant's reproductive output (Kim & Donohue, 2011). Understanding these is important for assessing the capacity of a population to persist under changing conditions. Critical abiotic factors include the availability of resources such as light, water, and nutrients, which may directly influence the numbers of flowers and fruits produced (Bateman, 1948). However, the response of plants to resource shortages is not only passive; for example, nutrient investment in reproduction may be prioritized (Lloyd, 1980) by selectively shedding damaged flowers, developing fruits, and/or genetically inferior seeds (Janzen, 1977).

The availability and quality of pollen are perhaps the most important biotic factors influencing reproductive success (Aizen & Harder, 2007; Wang, Zhang, Zhao, & Gadow, 2013). For example, seed set may be reduced if the pollen delivered to a flower is too closely (or too distantly) related to the mother plant (Bertin, 1982). The fragmentation of formerly continuous plant populations often disrupts plant–pollinator interactions because it reduces the range of pollen sources and the abundance and species diversity of pollinators (Steffan‐Dewenter & Tscharntke, 1999), which in turn may lead to reduced gene flow and seed set (reviewed in Knight et al., 2005). As a consequence, low‐density, fragmented plant populations often exhibit high levels of inbreeding and low fecundity (Severns, 2003).

The impact of these abiotic and biotic factors upon reproductive success is strongly influenced by evolutionary trade‐offs, such as those regulating pollinator attraction effort or the number and size of seeds (Haig & Westoby, 1988; Helenurm & Schaal, 1996). Large seeds may confer fitness benefits in shady habitats and on nutrient‐poor soils (Vaughton & Ramsey, 1998) because large seedlings are better able to survive under these conditions than seedlings of small‐seeded species (Moegenburg, 1996); on the other hand, smaller seeds can be produced in larger numbers and are more readily dispersed (Harper, Lovell, & Moore, 1970). Seed size appears to be under strong stabilizing selection, since it varies much less than vegetative structures such as leaves (Harper et al., 1970): in some species, however, seed size is rather variable (Thompson, 1984). Factors known to affect seed mass include resource constraints (Wulff, 1986), seed number and pollen availability (Wolf, Reed Hainsworth, Mercier, & Benjamin, 1986), position of the inflorescence, and position of the flower within the inflorescence (Winn, 1991), as well as different combinations of these factors (Galen, Plowright, & Thomson, 1985).

**Figure 1:**
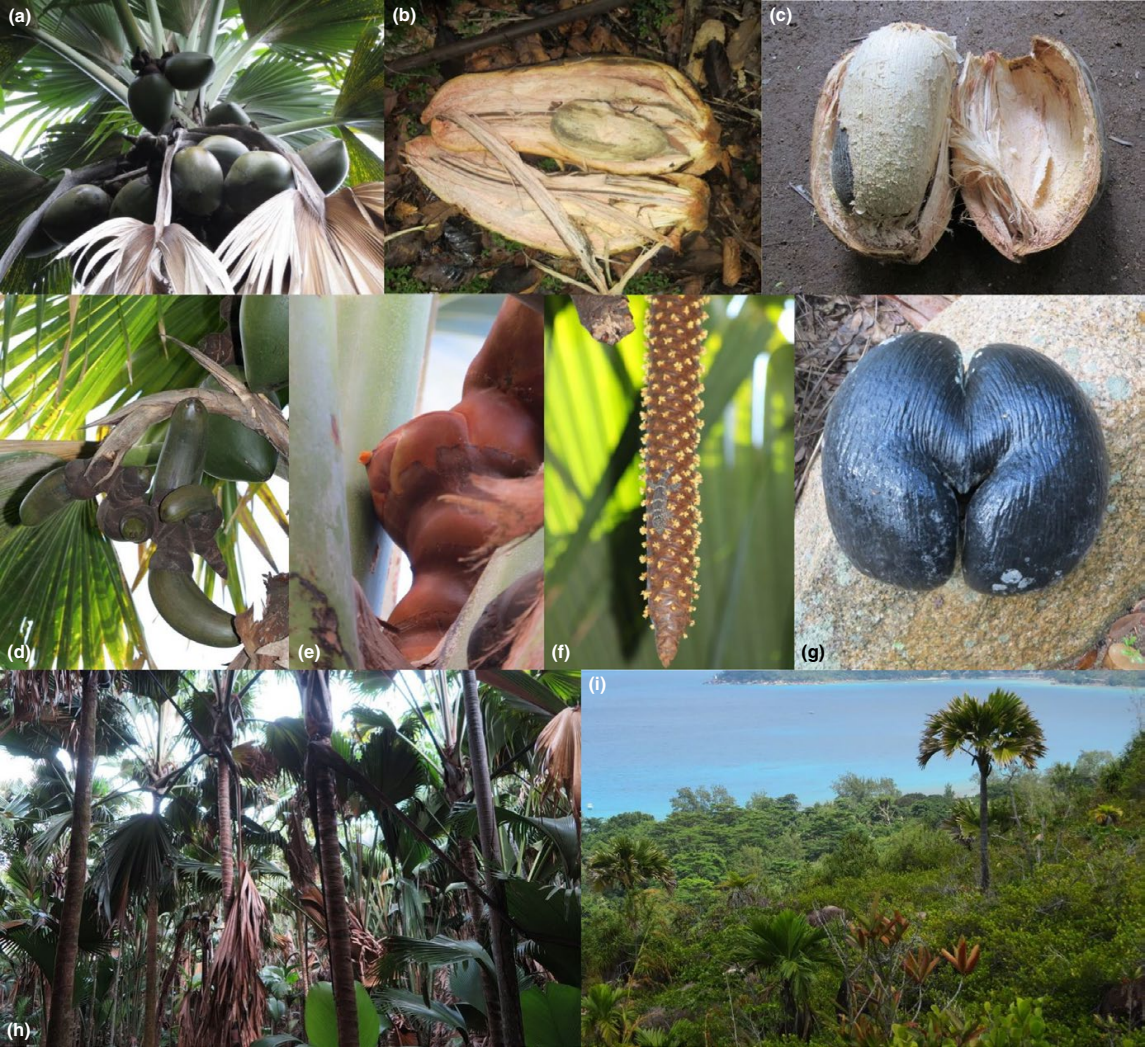
Photographs of *Lodoicea maldivica* on Praslin. (a) Female bearing a large fruit set. The most recently produced fruits can be observed on the uppermost inflorescences, and successively more mature fruits can be seen on inflorescences hanging lower down on the palm. (b) Dissected abnormal fruit. (c) Dissected fruit with seed inside. (d) Female bearing fruits and abnormal fruits. (e) Receptive female flower. (f) Gecko (*Ailuronyx trachygaster*) feeding on the nectar of a male inflorescence. (g) Seed. (h) Closed forest in Vallée de Mai. (i) Degraded shrubland in the north of Praslin. Two adult males can be observed amongst the shrub

By far the largest seeds in the plant kingdom are those of the Seychelles coco de mer *Lodoicea maldivica* (J. F. Gmel.) Pers. (Arecaceae), which may weigh as much as 18 kg (fresh weight; Figure 1a,c,g). Not surprisingly, most trees produce only a few seeds; a survey in the UNESCO World Heritage Site at the Vallée de Mai (VdM), the finest remaining stand of *Lodoicea* in the Seychelles, found that the mean number of developing fruits per female was 7.03, though with considerable variation among trees (Edwards, Kollmann, & Fleischmann, 2002). Given that it takes about 7 years for the fruits to mature (comments in Blackmore et al., 2012; Corner, 1966; Anders Lindström personal observation from Nong Nooch Tropical Botanical Garden, Thailand), and most fruits contain only one seed, this represents a reproductive output of only around one seed per female tree per year.

In its native habitat, *Lodoicea* has to cope with harsh environmental conditions, including deep shade for the early decades of its life, and very low levels of most soil nutrients. In these soils, the average reproductive output represents a considerable investment in terms of nutrients, accounting for some 88% of a female tree's annual aboveground phosphorus (P) budget (Edwards, Fleischer‐Dogley, & Kaiser‐Bunbury, 2015). Indeed, *Lodoicea* exhibits a remarkable mechanism to improve its nutrient supply by funnelling any nutrient‐rich material, especially pollen, falling on its leaves to the base of the trunk (Edwards et al., 2015). Geological evidence suggests that *Lodoicea* evolved in the absence of major disturbances over a period of some 70 million years (Baker & Miller, 1963), and we might therefore expect the main drivers for its reproductive success in a monodominant forest to be well established.

The extraordinary biological features of *Lodoicea* are the reason for its substantial contribution to the Seychelles economy. Around 40% of all tourists visiting the Seychelles pay an entrance fee for the VdM primarily to see the *Lodoicea* palm forest ecosystem, and considerable additional revenue is generated from the sale of the double coconuts to tourists (Seychelles Islands Foundation, 2009 unpublished report). To meet this demand, most seeds are collected, even from forest that is otherwise protected. It has been observed that trees growing alone or in small groups in degraded shrubland habitat (Figure 1i) usually produce fewer seeds than trees growing in closed forest (Figure 1h) (Edwards et al., 2015). Given the ecological and economic importance of the species, its unique life history and the degradation of palm forest habitat, it is critical to understand the processes that are responsible for this reduced fecundity.

The aim of this study was to investigate variation in flower and fruit production of *Lodoicea* growing in closed palm forest and in degraded shrubland, and to determine the main factors influencing this variation. Specifically, we asked the following questions: (1) “Are soil nutrients associated with inflorescence and flower number, and fruit set?” (2) “What is the relationship between pollen availability and healthy and abnormal fruit production?” (3) “What is the relationship between genetic diversity and flower and fruit production?” and (4) “Are habitat fragmentation and reduced adult tree density related to female fecundity?” In addition to exploring variation in numbers of flowers and fruits, we present data on seed size in *Lodoicea*. We discuss the implications of our results for the future sustainable management of *Lodoicea* and drivers of plant reproduction in general.

## 2 MATERIALS AND METHODS

### 2.1 Study area

**Figure 2:**
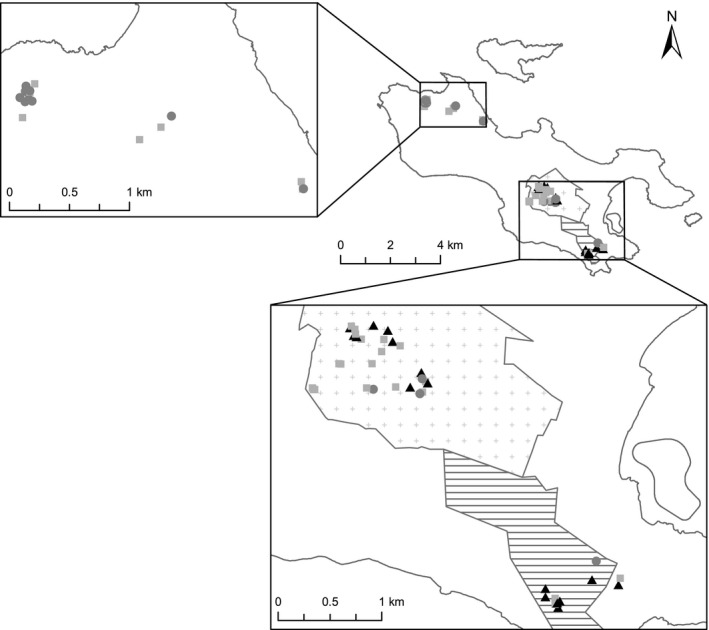
Locations of the sites of sampled female *Lodoicea maldivica* on Praslin. Black triangles are individuals that had six or more fruits; dark gray circles are those with no fruits; light gray squares are all others. Trees sampled in the south of the island belong to closed forest (*crossed area* = Praslin National Park, *lined area =* Ravin de Fond Ferdinand Nature Reserve), and trees sampled in the north belong to patches of *Lodoicea* trees in an otherwise homogeneous, degraded shrubland

*Lodoicea maldivica* is endemic on the island of Praslin (37.4 km^2^) and its satellite island Curieuse (3.5 km^2^) in the Republic of Seychelles. Until the 19th century, dense monospecific stands of *Lodoicea* covered much of the islands (Fauvel, 1915). Today, relatively undisturbed *Lodoicea* forest remains only in protected areas (Vallée de Mai and Fond Peper within Praslin National Park, *and* Ravin de Fond Ferdinand Nature Reserve*)* in the south of Praslin (Figure 2). On Curieuse and elsewhere on Praslin, the species persists as mainly small clusters and isolated individuals, with poor natural regeneration. *Lodoicea* kernel is CITES‐listed and protected from illegal exploitation (Kaiser‐Bunbury, Fleischer‐Dogley, Dogley, & Bunbury, 2014), although many nuts are poached due to their high value in the black market (Rist et al., 2010).

Fieldwork on the island of Praslin was conducted in the two main habitat types where *Lodoicea* occurs*:* dense, closed palm forest in the south, and open shrubland and mixed forest with scattered *Lodoicea* in the north (Figure 2). Praslin has a tropical humid climate, with a mean daily rainfall of 10.6 ± 1.1 mm and 17.1 ± 1.2 mm in the dry and wet seasons, respectively (Edwards et al., 2015). The highly weathered granitic soils are infertile and deficient in nitrogen (N), P, potassium (K), calcium, and magnesium (Dobrovol'skiy, 1986), particularly in eroded or rock‐strewn areas with only a skeletal soil.

### 2.2 Method for assessing female flower and fruit production

In closed forest on Praslin, trees reach sexual maturity when the trunk is about 4 m tall (Savage & Ashton, 1983; estimated ca. 25 years, Blackmore et al., 2012), although in open sites this may happen when the trunk is shorter. The male trees bear one to four long‐lived (3–4 months), cylindrical inflorescences up to 90 cm long that produce spirally arranged clusters of flowers (Figure 1f). Female plants bear large lignified inflorescences that are produced toward the stem apex in the axils of emerging leaves (Figure 1a). As each inflorescence grows in length, up to 13 large flowers are produced sequentially over a period of three to four weeks (C. Kaiser‐Bunbury personal observation; Figure 1e). After pollination, ovules expand rapidly over 5–6 months to reach their final size, and then develop into mature seeds over a period of 6–7 years. Each seed is surrounded by a hard shell, the pyrene, which is formed from maternal tissue (pericarp; Romanov, Bobrov, Wijesundara, & Romanova, 2011). The fruit consists of a thick husk (also formed from the pericarp), and usually contains a single seed, though some (9.2%) contain two seeds, and a very few (0.03%) contain three (*N *= 307, own data, trees on Praslin and Curieuse). Unfertilized ovules become lignified and persist as prominent, hemispherical lumps on the inflorescence. Some fruits fail to develop normally, being narrow and elongated in shape (Figure 1b,d), and are shed before reaching maturity. The reasons for this abnormal development are not known. We included abnormal fruits in our survey to assess how abnormal fruit production affects fecundity in different types of habitat.

The massive, woody inflorescences of female trees live for 7 years (i.e., until the fruits mature) and provide a record of the tree's reproductive history over this period (see TextS1 for method assumptions and limitations). Because each inflorescence is produced in the axil of a leaf, their order up the trunk represents the sequence in which they were produced. By examining the oldest inflorescence, it is possible to determine how many flowers were produced seven years previously, how many ovules were fertilized, and how many of these developed normally. The presence of a distinctive bowl‐shaped scar surrounded by lignified perianth parts shows that a mature fruit has already been shed. Occasionally a similar scar occurs on a younger inflorescence (i.e., one with still maturing fruits), which indicates that an immature fruit has been shed.

### 2.3 Field survey

We used the method described above to study the reproductive output of 57 female *Lodoicea* trees, chosen to represent varying degrees of isolation from male trees. To achieve a balanced representation of female trees along a gradient of distance to the nearest male, females were randomly selected within distance classes. Thirty‐nine of the trees were in palm forest, while 18 were in degraded shrubland. Trees with obvious signs of poaching were excluded from the study.

For each tree, we examined all inflorescences, recording the numbers of undeveloped ovules, developing fruits, abnormal fruits, and successfully shed mature fruits. We then aggregated these data to obtain “all flowers” (the sum of unfertilized flowers plus normal plus abnormal fruits), abnormal fruits, and “all fruits” (the sum of all developing fruits plus any mature fruits that had been shed). “Fruit set” was calculated as the proportion of “all fruits” to “all flowers.”

To test whether pollen availability and fruit production were related, we recorded for each female tree the distance to the nearest male *Lodoicea*, and the total number of males within a 10 m radius. This radius was chosen because a previous study showed that male and female pairs within 10 m from each other are significantly related (Morgan, Kaiser‐Bunbury, Edwards, Fleischer‐Dogley, & Kettle, 2017). Distances from females to the nearest male ranged from 0.4 to 159 m, and the numbers of males within a 10 m radius ranged from 0 to 9. *Lodoicea* have a long life span (up to 350 years according to one estimate; Savage & Ashton, 1983), making it unlikely that adult male densities would have changed greatly during the seven‐year period covered by our data. The number of flowering catkins per male (recorded between May and July 2014) ranged from 0 to 4 (mean ± *SD*: 0.67 ± 0.03; *N* = 320). Using 12 microsatellite loci developed by Morgan et al. (2016), we determined the genotypes of all females in our sample, from which we calculated their standardized multilocus heterozygosities (MLH; following Slate et al., 2004). These values were used to investigate any link between genetic variability and reproductive success.

### 2.4 Soil nutrient status around female trees

#### 2.4.1 Available P and K, and pH

To test whether flower production was associated with soil nutrient availability we collected samples of soil at 10 cm depth at distances of 0.5 and 1 m downhill from each female tree in April to May 2014. Means of both measurements were used for determining available P and K concentrations and pH (Edwards et al., 2015). Sites with insufficient soil were omitted. The samples were passed through a 2‐mm sieve, air‐dried, and extracted in a solution of ammonium acetate and EDTA (1:10; FAL, FAC, & RAC, 1996). The extracts were then analyzed using inductively coupled plasma optical emission spectroscopy (Vista‐MPX CCD Simultaneous ICP‐OES; Varian). Each ICP‐OES run included sample blanks and an external reference sample. Soil pH was determined in a 1:2.5 soil to distilled water solution using a portable pH meter (Microprocessor pH 95 Meter, WTW, Weilheim, Germany).

#### 2.4.2 Available N

Nitrogen (N) availability was measured by placing small mesh bags containing 2.0 g (dry weight) ion‐exchange resin (Amberlite IRN‐150, H^+^ & OH^−^ form; Sigma‐Aldrich Logistik GmbH, Schnelldorf, Switzerland) in the soil (IER; Lundell, 1989). The 5 × 5 cm bags were made from fine nylon mesh (60‐μM mesh width, Sefar Nitex 03‐60/35; Sefar AG, Thal, Switzerland). Prior to use, the bags were shaken for 2 h with 2 M KCl, rinsed with distilled water, and then kept moist until use. The resin bags were set out in the field by cutting an oblique slot in the soil to a depth of 5 cm, inserting the bag, and gently pressing back the soil. Bags were installed at distances of 0.5 and 1 m downhill from females, and incubated in the field for ~ 30 days. Mean daily rainfall during the incubation period was 8.2 ± 2.2 ml/day (within the normal range for the time of year). Upon collection the bags were rinsed with distilled water to remove surface soil and then air‐dried. In the laboratory, the resin was extracted for 2 h in 30 ml 2 M KCl (Keeney & Nelson, 1982). The extract was filtered through Whatman no. 45 filter paper and analyzed using colorimetric assays for ${\text{NH}}_{4}^{+}$ (adapted from Mulvaney, 1996) and ${\text{NO}}_{3}^{-}$ (plus ${\text{NO}}_{2}^{-}$ ; Doane & Horwáth, 2003; see Appendix 1 for detailed methods).

### 2.5 Statistical analysis

Pairwise correlations were used to determine the relationships between response variables and potential predictors prior to inclusion in the models. We used five different Generalized Linear Models (GLMs) and three functions (indicated below as “package::function()”) in the RStudio environment v. 0.98.1102 (RStudio Team, 2015). Co‐linearity of variables was tested using usdm::vifstep() (Naimi, 2015) by calculating the variance inflation factors (VIFs). All variables had VIF values below the recommended threshold value of 10 (max. VIF = 2.08), indicating no collinearity.

#### 2.5.1 Inflorescence and flower production

To test the influence of soil nutrients, pH, MLH, and vegetation type on the production of inflorescences and flowers, we modeled inflorescence and flower number as a function of the main effects N, P, K, pH, MLH, and vegetation type (dense closed forest or degraded shrubland), along with the following two‐way interactions in the full model: N × P, N × K, P × K, P × pH, K × pH, and vegetation type × MLH. Number of inflorescences was analyzed using a GLM assuming a Poisson distribution and log link. Flower number was analyzed using a GLM assuming a negative binomial distribution and a log link, correcting for overdispersion (MASS::glm.nb(); Venables & Ripley, 2002).

#### 2.5.2 Fruit production

To analyze the effects of pollen availability, MLH and vegetation type on fruit production, we used a model that included four main effects (distance to the nearest male, number of males within a 10 m radius, MLH and vegetation type), and also the following two‐way interactions: distance to the nearest male × vegetation type, distance to the nearest male × MLH, number of males within 10 m × vegetation type, number of males within 10 m × MLH and vegetation type × MLH. As a high proportion of trees bore no fruits (i.e., the fruit set data were zero‐inflated), we first ran a binary model (i.e., fruit‐setting probability), modeling the occurrence of successes (fruits > 0) and failures (fruits = 0), followed by a “proportional” model (i.e., fruit set size) on non‐zero data. For the binary model we used a GLM with a binomial distribution (across all populations, and within closed forest separately). The proportional data (from both vegetation types) were analyzed using a quasi‐binomial distribution to account for overdispersion. We used the cbind() function to link the numbers of flowers that did and did not develop into fruits, which accounts for the total number of flowers on a tree, thereby considering unbalanced data in the analysis. Bivariate correlations showed that availability of soil nutrients was not directly related to fruit set (see Table 1), which justified the exclusion of nutrients from the main fruit production models. To test for indirect effects between explanatory variables and fruit production we ran the same models as above with the additional main effects N, P, and K (see TableS1). Between 2009 and 2013, freshly fallen seeds from the VdM and Fond Peper were weighed (*N *= 2,416), and their lengths and diameters measured (*N* = 2,368; Seychelles Islands Foundation, unpublished data).

**Table 1:** Variables tested in this study for each female *Lodoicea maldivica*, including resin adsorption rates for nitrogen (N; ${\text{NH}}_{4}^{+}$ , and ${\text{NO}}_{3}^{-}$ combined), available soil phosphorus (P) and potassium (K), and soil pH (all measurements combined from 0.5 and 1 m sampling distances from females). Also measured were the distance to the nearest male and number of males within 10 m from the female, and the standardized multilocus heterozygosity (MLH) of the female. Spearman's *rho* correlation coefficients (except inflorescence number against soil pH and MLH, which were tested with Pearson's correlations), and significance levels are given. One outlying female that produced 43 fruits was excluded from all correlations

Variable	*N*	Mean (*SD*)	Range	Correlation coefficient			
				Inflorescence no.	Flower no.	Fruit no.	% fruit set
Available N (μg N/g/day)	56	4.90 (6.31)	0.46–28.79	0.317	0.381**	0.002	−0.090
Soil P (μg P/g dry soil)	52	3.72 (3.20)	0.31–14.99	0.315	0.196	0.164	0.126
Soil K (μg K/g dry soil)	52	129.35 (97.92)	31.00–509.05	0.370	0.235	0.241	0.166
Soil pH	52	4.93 (0.43)	3.76–6.34	0.267	0.158	0.094	0.069
Distance to nearest male (m)	57	28.06 (34.87)	0.4–159	0.066	0.159	−0.483***	−0.529****
No. males ≤ 10 m	57	0.93 (1.69)	0–9	−0.143	−0.240	0.465***	0.533****
MLH	57	0.768 (0.243)	0.360–1.321	−0.073	−0.041	0.020	−0.023

****P ≤ .0001, ***P ≤ .001, **P ≤ .01 Significance values after sequential Bonferroni corrections for each response variable.

#### 2.5.3 Abnormal fruit production

To study the effects of nutrients, pollen availability, MLH, and vegetation type on the occurrence of abnormal fruits, we modeled the abnormal fruit as a function of N, P, K, distance to the nearest male, number of males within 10 m, vegetation type and MLH, and the two‐way interactions: N × P, N × K, P × K, N × vegetation type, K × vegetation type, P × vegetation type, distance to the nearest male × MLH, number of males within 10 m × MLH, and vegetation type × MLH. The data on abnormal fruits were modeled using the occurrence of successes (abnormal fruits > 0) and failures (abnormal fruits = 0) assuming a quasi‐binomial distribution with a logit link to account for overdispersion.

#### 2.5.4 Model selection

We applied a backward stepwise model selection for all GLMs to obtain minimum adequate models. For the Poisson, negative binomial and binomial models, we excluded variables using Akaike's second order information criterion (AICc; for smaller sample sizes). For the quasi‐binomial models we used the function MuMIn::QAIC() (Barton, 2016) for model simplification based on the quasi‐AICc values. The removal criterion—Δ(Q)AICc ≤ 2 compared to the reduced model—was selected to ensure a parsimonious model selection and avoid overfitting (Arnold, 2010). Main effects were only removed when higher‐order effects were removed first.

## 3 RESULTS

### 3.1 Inflorescences and flowers

Female trees within closed forest and degraded shrubland produced similar numbers of inflorescences per tree (range 1–15, *t*_55_ = 0.309, *p *= .76) and flowers per inflorescence (range 2.4–9.1, *t*_55_ = 0.591, *p *= .56, Table 2). Similarly, the total numbers of female flowers per tree (i.e., all flowers produced over the previous 7 years; range 6–123) did not differ between vegetation types (*W* = 352, *p* = .99, Table 2). Numbers of inflorescences and flowers were both positively associated with available soil N and K, but not with P and pH (Table 3a,b; see also Table 1 and Fig.S1a–d, and TableS2 for results of all penultimate models). Soil nutrients and pH were highly variable, and similar in forest and degraded shrubland (Wilcoxon all *p *> .05).

**Table 2:** Variation in reproductive output of female *Lodoicea maldivica* included in this study. Reported are the means (±*SD*). Fruit set is defined as proportion of flowers that developed into fruits

Reproductive output	Closed forest (*N* = 39)	Degraded shrubland (*N* = 18)	Overall (*N *= 57)
No. of inflorescences	6.97 (3.00)	6.78 (2.56)	6.91 (2.85)
No. of flowers (all)/ inflorescence	5.26 (1.77)	4.95 (0.04)	5.16 (1.66)
No. of flowers (all)/ tree	39.62 (28.14)	35.72 (21.00)	38.39 (25.97)
No. of undeveloped ovules/tree	31.92 (24.99)	31.06 (17.68)	31.65 (22.78)
No. of fruits (all)/ tree	6.18 (7.27)	0.72 (1.02)	4.46 (6.54)
No. of developing fruits/tree	5.62 (6.80)	0.61 (1.04)	4.04 (6.10)
No. of fallen immature fruits/tree	0.36 (1.14)	0.00 (0.00)	0.25 (0.95)
No. of fallen mature fruits/tree	0.15 (0.43)	0.06 (0.24)	0.12 (0.38)
Fruit set	0.21 (0.19)	0.03 (0.04)	0.16 (0.18)
No. of abnormal fruits/tree	1.54 (4.53)	4.00 (6.37)	2.32 (5.25)

**Table 3:** Final GLM models for female *Lodoicea maldivica* fecundity

	Estimate	*SE*	*z* value	Pr(>|*z*|)
(a) Response: inflorescence number				
Intercept	1.6789	0.0988	16.998	<2e‐16***
N	0.0121	0.0077	1.577	0.1148
K	0.0012	0.0005	2.396	0.0166*
Null deviance: 58.065 on 50 *df*		ΔAICc (full & final): 19.6		
Residual deviance: 49.966 on 48 *df*		ΔAICc (penultimate & final): −0.2		
(b) Response: flower number				
Intercept	3.2506	0.1483	21.924	<2e‐16***
N	0.0209	0.0125	1.672	0.0944 .
K	0.0018	0.0008	2.173	0.0298*
Null deviance: 61.526 on 50 *df*		ΔAICc (full & final): 21.8		
Residual deviance: 53.194 on 48 *df*		ΔAICc (penultimate & final): 0.3		
(c) Response: Presence of fruit(s) (both vegetation types)				
Intercept	2.1691	0.5278	4.110	3.96e‐05***
Degraded shrubland	−2.1691	0.7076	−3.065	0.00218**
Null deviance: 61.210 on 56 *df*		ΔAICc (full & final): 12.3		
Residual deviance: 50.746 on 55 *df*		ΔAICc (penultimate & final): −1.3		
(d) Response: Presence of fruit(s) (closed forest)				
Intercept	2.1691	0.5278	4.11	3.96e‐05***
Null deviance: 25.793 on 38 *df*		ΔAICc (full & final): 5.7		
Residual deviance: 25.793 on 37 *df*		ΔAICc (penultimate & final): 2.0		
(e) Response: Fruit set when fruit(s) present (both vegetation types)				
Intercept	−0.64972	0.2258	−2.878	0.00639**
Distance to nearest male	−0.0690	0.0182	−3.781	0.00051***
Degraded shrubland	−2.1272	0.8579	−2.596	0.01312*
Distance to nearest male × degraded shrubland	0.0624	0.0214	2.920	0.00573**
Null deviance: 273.27 on 43 *df*		ΔQAICc (full & final): 9.1		
Residual deviance: 134.99 on 40 *df*		ΔQAICc (penultimate & final): 1.9		
(f) Response: Presence of abnormal fruit(s)				
Intercept	−2.2305	0.4968	−4.490	3.69e‐05***
Distance to nearest male	0.0507	0.0143	3.549	8e‐04***
Null deviance: 71.097 on 56 *df*		ΔQAICc (full & final): 10.3		
Residual deviance: 48.910 on 55 *df*		ΔQAICc (penultimate & final): −0.4		

***p ≤ .001, **p ≤ .01, *p ≤ .05, *p* < .01.

### 3.2 Fruits

**Figure 3:**
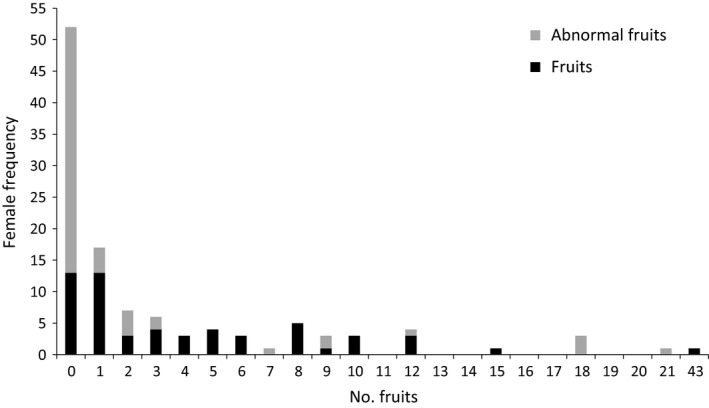
Frequency histogram showing numbers of fruits and abnormal fruits produced by individual female *Lodoicea maldivica* trees. Each female is represented twice: once each for the numbers of fruits and abnormal fruits

The numbers of developing and mature fruits per tree ranged from 0 to 43, yet the frequency distribution was highly skewed (median = 2). Thirteen trees (22.8%) produced no fruits and 17 (29.8%) produced six or more (fruits from these 17 accounting for 75% of all fruits recorded; Figure 3). The average number of fruits per inflorescence was 0.68 for a sample of 371 inflorescences, with 64% of inflorescences bearing no fruits. Except for a few small inflorescences with four or fewer flowers, it never happened that all flowers developed into fruits. Indeed, we found only two inflorescences bearing more than four fruits. As only 4% of inflorescences had scars indicating the former presence of a mature fruit, we conclude that the fruits on one inflorescence mature at approximately the same time, with the inflorescence being shed soon after the fruits have fallen (Table 2).

Trees in closed forest produced over 8 times as many fruits as those in degraded shrubland (Table 2, W = 607, *p* ˂ .0001), and the GLM models confirmed that presence of fruits was strongly associated with closed forest (Table 3c). As only seven trees in degraded shrubland had fruits, we did not perform any detailed analyzes to explain their presence. All tested variables were unsuitable to explain the presence of fruiting/nonfruiting trees in closed forest, where 87% of trees produced fruits over a 7‐year period (Table 3d). Fruit set, however, decreased with increasing distance to the nearest male, this pattern being more marked in closed forest than in degraded shrubland (Table 3e and Fig.S1e–f).

The number of flowers was independent of the number of fruits produced by individual fruit‐bearing females (outlier excluded, adj *R* ^*2* ^= −0.02, *p* = .66). Across all trees, and assuming a seven‐year maturation period for fruit, the mean rate of production in closed forest was 0.88 fruits/year compared to 0.10 fruits/year in degraded shrubland (overall 0.67 fruits/year; fruit‐bearing trees only, 0.98 vs. 0.21 fruits/year). Pairwise correlations indicated that the number of males within a 10 m radius of the female, as well as the distance to the nearest male significantly influenced fruit number and fruit set (Table 1).

### 3.3 Abnormal fruits

Over half of all fruits were of the abnormal, elongated type (51.2%; Figure 3). The percentage of abnormal fruits was much higher in degraded shrubland than in closed forest (range 0–21, 61% (11/18) vs. 18% (7/39) females with abnormal fruit, *W* = 201.5, *p* = .002, Table 2). Within closed forest, abnormal fruits were highly aggregated on certain females, with most trees having either only normal or only abnormal fruits (Fisher's two‐tailed exact test, *p *= .006; Table 4). However, no significant aggregation of abnormal fruits was found in degraded shrubland (Table 4). The probability of bearing abnormal fruits increased with distance from the nearest male (Table 3f). Inflorescences with abnormal fruits bore markedly more fruits (up to 9 abnormal fruits per inflorescence, often a mixture of normal and abnormal) than inflorescences with only normal fruits.

**Table 4:** Contingency table of female *Lodoicea maldivica* with fruits and abnormal fruits in closed forest, degraded shrubland and overall populations. Total numbers for each category are given in brackets. Fisher's two‐tailed exact probabilities are shown

Abnormal fruits	Fruits		Total
	+	−	
Closed forest^a^			
+	42.86% (3)	57.14% (4)	100%
−	93.75% (30)	6.25% (2)	100%
Total	84.62% (33)	15.38% (6)	(39)
Degraded shrubland^b^			
+	54.55% (6)	45.45% (5)	100%
−	42.86% (3)	57.14% (4)	100%
Total	50.00% (9)	50.00% (9)	(18)

a Two‐tailed exact test *p* = .006.

b Two‐tailed exact test *p* = 1.

### 3.4 Seed size and mass

**Figure 4:**
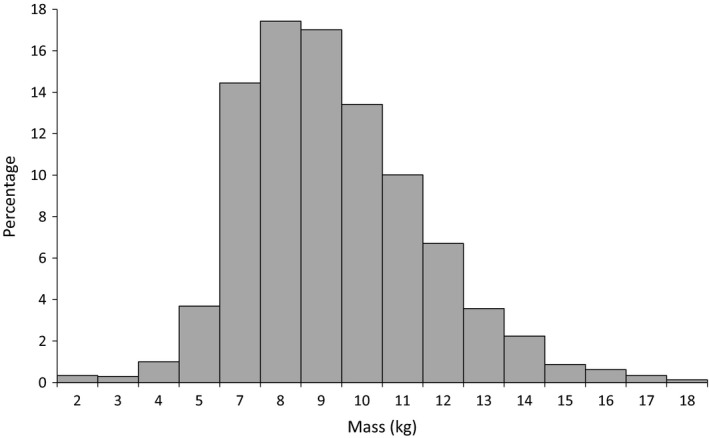
Frequency distribution of the fresh weights (kg) of *Lodoicea maldivica* seeds (*N *= 2,416)

Seeds varied greatly in size. Seeds collected over a 4‐year period showed a 16.3‐fold range in fresh weight, from 1.04 to 18 kg (mean ± *SD*: 8.50 ± 2.39 kg; *N* = 2415, Figure 4). Seed length and diameter (*N* = 2,368) ranged from 17 to 48 cm (mean ± *SD*: 29.57 ± 3.85 cm) and 12.2 to 40.6 cm (mean ± *SD*: 28.28 ± 3.87 cm), respectively (FigsS2 and S3).

## 4 DISCUSSION

### 4.1 Effects of soil nutrient and pollen availability

Our study shows that both soil nutrient and pollen availability influence the reproductive performance of *Lodoicea* growing on the nutrient‐poor soils in its native habitat on the island of Praslin. Soil N and K availabilities limit the total numbers of female inflorescences and flowers produced, and thus, set an upper limit to how many fruits a tree can bear. An earlier study on nutrient reabsorption rates in *Lodoicea* suggested that N and P are in very short supply (Edwards et al., 2015), but the significance of K as a limiting factor was previously unknown. In other palm species, including the economically important coconut *Cocos nucifera*, N shortage has been shown to limit female flower production and yield, K shortage to reduce fruit set and yield, and P shortage to restrict nut size (Smith, 1969).

Pollen availability appears to limit fruit set of *Lodoicea*, especially in open shrubland where the nearest male tree may be some distance away. Reproductive performance is further compromised because many fruits fail to develop normally, especially in degraded habitats. The reasons for this phenomenon are uncertain, but it could be due to a lack of compatible pollen.

As a consequence of these effects, female *Lodoicea* trees produce markedly more fruit in closed palm forest than when growing in open shrubland. Perhaps the most important reason for this difference is the proximity to the nearest male tree or trees. Although there is little information about pollination vectors, it is likely that endemic geckos, and perhaps also wind, play an important role (C. Kaiser‐Bunbury and Seychelles Islands Foundation, unpublished data). Two potential candidate gecko species, the day gecko (*Phelsuma sundbergi*) and the giant bronze gecko (*Ailuronyx trachygaster*), are specialized on *Lodoicea* forest, and rarely found or absent from degraded shrub vegetation (Noble, Bunbury, Kaiser‐Bunbury, & Bell, 2011; Seychelles Islands Foundation, unpublished data). The absence or smaller population sizes of *Lodoicea* pollinators, lower densities of male trees (Ågren, 1996), and the tendency of diecious plants to be pollen‐limited (Wilson & Harder, 2003) may all contribute to reduced pollination and consequently fruit set in degraded shrubland, despite similar soil nutrient levels in both habitats.

We found no evidence that either flower or fruit production was affected by genetic diversity of female trees. The effects of genetic diversity on plant fecundity are largely unknown, although heterozygosity has been shown to correlate with growth rates and survival in some species (Breed et al., 2012; Nutt et al., 2017). Inbreeding and outbreeding were not directly measured in our study, mainly because it takes as long as a decade from pollination to the production of the first leaf. Based on the outcome of our research, however, controlled fertilization and transplant experiments are planned. These experiments will also help establish more clearly whether pollen limitation is a factor limiting seed set in isolated plants.

### 4.2 Abnormal fruits

Although abnormal fruits were recorded on over half of all the trees studied, some individuals were much more affected than others. Abnormal fruits were more common in degraded shrubland, but the presence could not be attributed to differences in soil nutrients. Our data suggest that there is a strong spatial component in female trees for being prone to produce abnormal fruit, which may be due to pollination limitation. Several nonmutually exclusive hypotheses could explain the presence of abnormal fruits, including: parthenocarpy (i.e., the development of unfertilized fruit, which may or may not have been pollinated or otherwise stimulated to grow) due to a genetic effect (Gorguet et al., 2008), or stenospermocarpy (i.e., the abortion of fruit after fertilization of the ovule) due to disease (Berry, 1960), or inadequate resources (Lloyd, 1980). Alternatively, pollen received by females could have been too closely or too distantly related, or otherwise incompatible. Abnormal fruits never developed when sufficient, mixed and fresh pollen was applied by hand to viable female flowers in Fond Ferdinand (Terence Payet, pers. comm., Seychelles Islands Foundation). Dissections of two abnormal fruits from the Vallée de Mai revealed extensive growth of the maternal mesocarp tissue, but no evidence of biparental endosperm or embryonic tissue (Romanov et al., 2011). This suggests that pollination most likely occurred but fertilization failed (Mikhail Romanov pers. comm.). Based on our observations in the entire population, pollen limitation and genetic causes may be the most likely explanation, although further long‐term quantitative work and hand‐pollination experiments will be required to determine the exact cause and its consequences for fitness mechanisms. Whatever the cause, abnormal fruit production is substantially reducing seed production, especially in open areas where natural regeneration is most needed.

### 4.3 Regulation of fruit numbers

In addition to the effects of nutrient and pollen availability upon fruit production, our data suggest that following pollination *Lodoicea* may regulate the number of flowers that mature into fruits. This would explain why even large inflorescences with 10 or more flowers rarely bear more than four fruits, even in closed forest. This aspect of regulation probably depends upon the carbon balance of the tree, and operates through pollinated flowers becoming such strong carbon sinks that they suppress ovules that are pollinated later (Bangerth, 1989). Competition of this kind has been reported for many plants, including tropical trees, though more commonly amongst the ovules within a fruit rather than amongst flowers within an inflorescence (Mohan Raju, Uma Shaanker, & Ganeshaiah, 1996; Teixeira, Pereira, & Ranga, 2006).

The mechanisms regulating fruit numbers in *Lodoicea* appear to operate at an early stage, as only 5% of developing fruits were shed before reaching maturity. This would explain the unexpected negative relationship between fruit set and soil nutrients (see TableS1); females growing on nutrient‐rich soil may produce more flowers than those growing on poorer soil, but because the number that develop is regulated, a smaller proportion actually set fruit. Our data also suggest that if the recently pollinated ovule is abnormal, it does not suppress neighboring fruits, with the consequence that some inflorescences had as many as nine fruits. A similar effect has been reported for seedless cucumbers (Denna, 1973).

### 4.4 Variation in seed size

*Lodoicea* exhibits great plasticity in seed size, which appears to exceed that of any other palm (Moegenburg, 1996) or plant species (Thompson, 1984). One reason could be the large variation in the availability of nutrients that *Lodoicea* needs in large amounts to produce its seeds. Such an effect of nutrient availability upon seed mass has been demonstrated in *Banksia marginata,* growing in very poor soils in Australia (Vaughton & Ramsey, 1998). It is significant that *Lodoicea* has developed a remarkable mechanism to capture nutrients by funnelling them to the base of the trunk, thereby influencing the spatial distribution of nutrients in the forest (Edwards et al., 2015). However, in recent decades, trees have been planted without regard to soil nutrient conditions, which could partly explain the large contemporaneous variation in seed size.

## 5 DOES ANTHROPOGENIC HABITAT DISTURBANCE INFLUENCE FEMALE REPRODUCTIVE SUCCESS?

Anthropogenic forest degradation appears to have no effect on the production of inflorescences and flowers in *Lodoicea*, but greatly reduces fruit production, presumably because it causes pollen limitation. Even in closed forest, fruit production was probably higher in the past than it is today; for example, Ward (1866) reported that trees produced around four or five fruits per inflorescence and a maximum of 11, compared to the mean of 0.97 per inflorescence and maximum of 6 in our study. Many evolutionary theories predict that plants evolve to reduce pollen limitation, either by the attraction of pollinators (Haig & Westoby, 1988), the reduced reliance on pollinators (Lloyd, 1974) or the evolution of sexual reproductive traits (e.g., monoecy). *Lodoicea* has certainly evolved extraordinary sexual dimorphism, with a high reliance on pollinators with small home ranges, suggesting that the recently reduced and fragmented *Lodoicea* populations (Lionnet, 1976) are little resilient to man‐made habitat degradation and population thinning. Fragmentation caused by forest clearance and fires not only reduced the numbers and densities of reproductive adults, but also adversely affected *Lodoicea*'s pollinators, some of which are habitat‐endemics to *Lodoicea* forests.

## 6 MANAGEMENT RECOMMENDATIONS

Our results suggest that the first aim of management should be to restore closed *Lodoicea* forest conditions wherever possible, as females produce more fruits and fewer abnormal fruits under these conditions. Therefore, fruit should not be collected and translocated for restoration planting across extensive degraded areas, as was previously carried out on Curieuse Island in the early 2000s (Fleischer‐Dogley, 2006). The conservation and promotion of local pollinator communities will also be crucial for reducing future pollen limitations and increasing fruit production. It is thought that monodominant forests such as those of *Lodoicea* could only have evolved under relatively stable conditions over a very long‐time period (Hart, Hart, & Murphy, 1989) and thus are likely to be particularly sensitive to ecological perturbations. Fragmentation of these forests, which can result in reduced female fecundity, may have important evolutionary consequences, as the processes maintaining the species' dominance are disrupted. Future controlled pollination experiments will be highly relevant in guiding management strategies, and understanding the role of pollinators and relatedness levels of parent trees in fruit and abnormal fruit production.

## DATA ACCESSIBILITY

Data available from the Dryad Digital Repository: https://doi.org/10.5061/dryad.s4k1q.

## AUTHOR'S CONTRIBUTIONS

E.J.M., C.N.K‐B., P.J.E., and C.J.K. conceived the ideas and designed methodology. F.F‐D. made field work possible. E.J.M. conducted the field and laboratory work and analyzed the data. C.N.K‐B., P.J.E., F.F‐D., and C.J.K. provided advice and input to field work. C.N.K‐B., P.J.E. and C.J.K. supervised data analysis. E.J.M., C.K.N‐B., P.J.E., and C.J.K. led the writing of the manuscript, with contributions from F.F.‐D. All authors gave final approval for publication.

## CONFLICTS OF INTEREST

There are no conflicts of interest to declare.

## APPENDIX 1 PREPARATION OF REAGENTS

### ${\text{NH}}_{4}^{+}$ salicylate reagent

The salicylate reagent solution to assay for ${\text{NH}}_{4}^{+}$ was prepared by adding 0.05 g sodium nitroprusside, 13 g sodium salicylate, 10 g sodium citrate, and 10 g sodium tartrate to 100 ml dH_2_O. The hypochlorite reagent was made by dissolving 6 g sodium hydroxide in 100 ml dH_2_O and 2 ml sodium hypochlorite. About 200 µl each reagent were added to 800 µl sample in cuvets. After 60 min, absorbance was determined at 650 nm using a V‐1200 Spectrophotometer (VWR International GmbH, Dietikon, Switzerland), and readings compared to standard solutions (0–3 ppm; VWR International GmbH). Sample filtrates were diluted with 2 M KCl to give values in the linear range of absorbency.

### NO_3_^−^ vanadium reagent

The vanadium reagent to assay for NO_3_^−^ was prepared by dissolving 0.5 g vanadium (III) chloride, 0.2 g sulfanilamide, and 0.01 g N‐(1‐naphthyl)ethylenediamine dihydrochloride in 200 ml 0.5 M HCl. About 1,000 µl reagent was added to 45 µl sample in cuvets. Absorbencies were read at 540 nm after 6 h, and regressed against standard solutions (0–30 ppm; VWR International GmbH). Sample filtrates were also diluted when necessary. N adsorption rates were calculated (µg N/g resin/day; hereafter referred to as N), using the means of the 0.5 and 1 m sampling distances from the females.
