# Supplementary material for: Tracing coco de mer's reproductive history: Pollen and nutrient limitations reduce fecundity
Source: Ecol Evol. 2017 Aug 24;7(19):7765–76. doi: 10.1002/ece3.3312 (PMC5632624; doi:10.1002/ece3.3312)
Supplement: Supplementary file 1 [file ECE3-7-7765-s001.DOCX]

**Supporting Information**

**Text S1** Assessing female *Lodociea maldivica* reproductive success**.**

Various lines of evidence suggest that inflorescences persist for around seven years. First, we found very few older inflorescences bearing scars of fallen mature fruits, indicating that inflorescences are shed when the fruits have matured. Second, our method yields a mean rate of mature fruit production of 0.88 fruits/year in closed forest, which is similar to that estimated from a survey of all trees in the Vallée de Mai (VdM; 1.00 fruit per tree, Edwards, Kollmann & Fleischmann 2002), and to that estimated by the productivity of female trees (0.8 fruit per year between 1995 and 2000, Fleischer-Dogley 2006). Finally, the value corresponds well with results from a study of the rate of leaf production, which found that trunked immature *Lodoicea* in the VdM produced between 0.62 and 2.18 leaves per year (mean value 1.3 leaves per year, *N* = 15; Edwards, Fleischer-Dogley & Kaiser-Bunbury 2015). Since one inflorescence is produced per leaf, we would expect trees to bear somewhere between four and 15 inflorescences (mean ± SD = 9.4 ± 3.2), which corresponds closely to what we observed during this study.

A possible limitation of this assumption would be if it did not apply for inflorescences bearing only unfertilised ovules or a mixture of unfertilised ovules and abnormal fruits. We might expect these inflorescences to be shed sooner, and our analysis would then overestimate the proportion of ovules producing mature fruits. There is, however, some evidence to suggest that such an effect, if it exists, is probably small; our results show that just over half (54.5%) of individuals with fruit-bearing inflorescences also had older inflorescences with only unfertilised flowers, indicating that even these unfertilised inflorescences may persist for many years. We therefore base our analysis on the assumption that all inflorescences remain on the plant for the same time span of approximately seven years, although further research is needed to determine a more accurate estimate.

**Table S1** GLM models for female *Lodoicea maldivica* fecundity. Effects of nutrients are included.

|  | Estimate | SE | z value | | Pr(>\|z\|) |
| --- | --- | --- | --- | --- | --- |
| 1. **Response: Presence of fruit(s) (both vegetation types)** | | | | | |
| Intercept | 1.6799 | 0.8886 | 1.891 | 0.0587 . | |
| Degraded shrubland | -2.9540 | 0.8713 | -3.390 | 0.0007 *** | |
| N | 0.2295 | 0.1870 | 1.227 | 0.2197 | |
| P | -0.0030 | 0.1334 | -0.023 | 0.9818 | |
| Null deviance: 55.651 on 50 df ΔAICc (full & final): 18.8 | | | | | |
| Residual deviance: 36.558 on 47 df ΔAICc (penultimate & final): 1.2 | | | | | |
| 1. **Response: Presence of fruit(s) (closed forest)** | | | | | |
| Intercept | 2.1691 | 0.5278 | 4.11 | 3.96e-05 *** | |
| Null deviance: 25.793 on 38 df ΔAICc (full & final): 13.2 | | | | | |
| Residual deviance: 25.793 on 38 df ΔAICc (penultimate & final): 0.7 | | | | | |
| 1. **Response: Fruit-set when fruit(s) present (both vegetation types)** | | | | | |
| Intercept | -0.0218 | 0.3071 | -0.071 | 0.9439 | |
| Distance to nearest male | -0.0711 | 0.0180 | -3.949 | 0.0004 *** | |
| Degraded shrubland | -3.4670 | 1.3002 | -2.666 | 0.0118 * | |
| N | -0.0333 | 0.0183 | -1.825 | 0.0771 . | |
| P | -0.0933 | 0.0401 | -2.326 | 0.0263 * | |
| Distance to nearest male × degraded shrubland | 0.0730 | 0.0228 | 3.204 | 0.0030 ** | |
| Null deviance: 252.75 on 38 df ΔQAICc (full & final): 9.1 | | | | | |
| Residual deviance: 100.21 on 33 df ΔQAICc (penultimate & final): 1.9 | | | | | |

*** *P* ≤ 0.001, ** *P* ≤ 0.01, * *P* ≤ 0.05, . *P* < 0.01.

**Table S2** GLM model comparisons for female *Lodoicea maldivica* fecundity. Final and penultimate models (final-1) are shown, plus third (final-2) and fourth (final-3) last models when ΔAICc < 2 compared to final model.

| Model | Variables | AICc | ΔAICc |
| --- | --- | --- | --- |
| **Response: inflorescence number** | | | |
| Final | N + K | 244.5331 |  |
| Final-1 | N + K + pH | 244.3338 | -0.1993 |
| Final-2 | N + K + pH + vegetation | 246.0199 | 1.4868 |
| **Response: flower number** | | | |
| Final | N + K | 453.2447 |  |
| Final-1 | N + K + pH | 453.582 | 0.3373 |
| **Response: Presence of fruit(s) (all populations)** | | | |
| Final | Vegetation | 54.96861 |  |
| Final-1 | Distance + vegetation | 56.95824 | 1.98963 |
| **Response: Presence of fruit(s) (closed forest)** | | | |
| Final | - | 27.9012 |  |
| Final-1 | Distance | 28.55642 | 0.65522 |
| Final-2 | Distance + no. males | 27.20688 | -0.69432 |
| Final-3 | Distance + no. males + MLH | 29.66785 | 1.76665 |
| **Response: Fruit-set when fruit(s) present (all populations)** | | | |
| Final | Distance + vegetation + distance:vegetation | 82.92477* |  |
| Final-1 | Distance + vegetation + distance:vegetation + no. males | 84.58673* | 1.66196* |

*QAICc, “:” indicates an interaction term. Vegetation = vegetation type (closed forest/degraded shrubland); MLH = standardised multilocus heterozygosity; distance = distance to nearest male (m); no. males = number of males within 10 m.

**
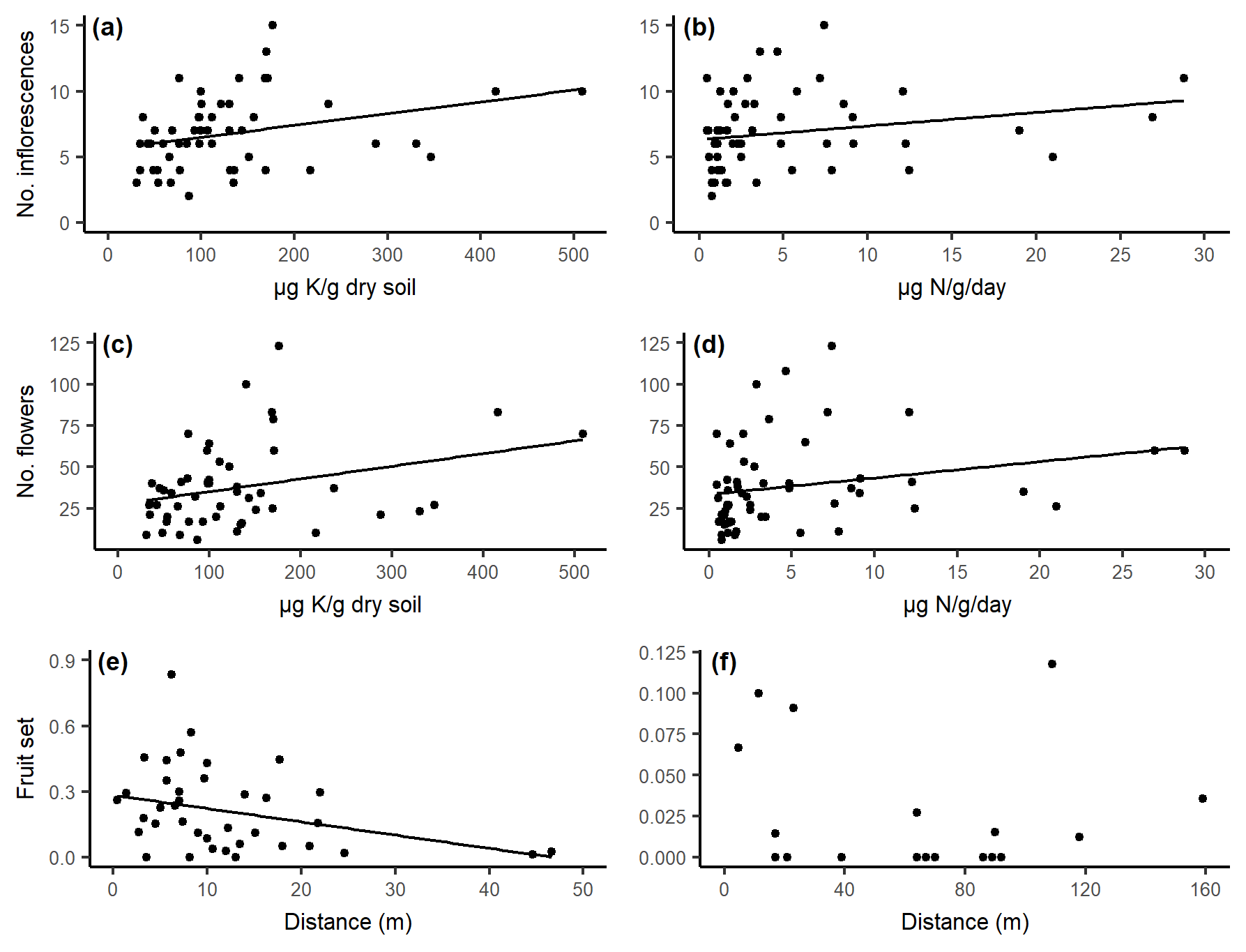
**

**Fig. S1** Linear relationships between female *Lodoicea maldivica* reproductive output and environmental variables. **(a)** Inflorescence number vs. available soil K. **(b)** Inflorescence number vs. available soil N. **(c)** Flower number vs. available soil K. **(d)** Flower number vs. available soil N. **(e)** Fruit-set vs. distance to the nearest male tree in closed forest. **(f)** Fruit-set vs. distance to the nearest male tree in degraded shrub land. Lines of best fit shown for significant correlations, all *P* < 0.05.

**Fig. S2** Frequency distribution of the lengths (cm) of *Lodoicea maldivica* seeds (*N* = 2368).

**Fig. S3** Frequency distribution of the diameters (cm) of *Lodoicea maldivica* seeds (*N* = 2368).

**References**

Edwards, P.J., Fleischer-Dogley, F. & Kaiser-Bunbury, C.N. (2015) The nutrient economy of *Lodoicea maldivica*, a monodominant palm producing the world’s largest seed. *New Phytologist,* **206**, 990–999.

Edwards, P.J., Kollmann, J. & Fleischmann, K. (2002) Life history evolution in *Lodoicea* maldivica (Arecaceae). *Nordic Journal of Botany,* **22**, 227–237.

Fleischer-Dogley, F. (2006) *Towards sustainable management of Lodoicea maldivica (Gmelin) Persoon*. PhD Thesis, The University of Reading, Reading, United Kingdom.
